# Association Between Tumor Mutation Burden (TMB) and Outcomes of Cancer Patients Treated With PD-1/PD-L1 Inhibitions: A Meta-Analysis

**DOI:** 10.3389/fphar.2019.00673

**Published:** 2019-06-14

**Authors:** Jiaxin Zhu, Tiantian Zhang, Jiahao Li, Junming Lin, Wenhua Liang, Wenjie Huang, Ning Wan, Jie Jiang

**Affiliations:** ^1^College of Pharmacy, Jinan University, Guangzhou, China; ^2^The First Affiliated Hospital of Jinan University, Guangzhou, China; ^3^International Cooperative Laboratory of Traditional Chinese Medicine Modernization and Innovative Drug Development of Chinese Ministry of Education (MOE), Jinan University, Guangzhou, China; ^4^Department of Thoracic Surgery and Oncology, the First Affiliated Hospital of Guangzhou Medical University, Guangzhou, China; ^5^Guangzhou Institute of Respiratory Disease & China State Key Laboratory of Respiratory Disease & National Clinical Research Center for Respiratory Disease, Guangzhou, China; ^6^Department of Respiratory Medicine, General Hospital of Southern Theatre Command, Guangzhou, China; ^7^Department of Pharmacy, General Hospital of Southern Theatre Command, Guangzhou, China; ^8^Guangzhou Huabo Biopharmaceutical Research Institute, Guangzhou, China; ^9^Dongguan Institute of Jinan University, Dongguan, China

**Keywords:** tumor mutation burden, cancer, PD-1/PD-L1 inhibitions, biomarker, meta-analysis

## Abstract

**Background:** Programmed cell death 1 (PD-1) or programmed cell death ligand 1 (PD-L1) inhibitions are being strongly recommended for the treatment of various cancers, while the efficacy of PD-1/PD-L1 inhibitions varies from individuals. It is urgent to explore some biomarkers to screen the most appropriate cancer patients. Tumor mutation burden (TMB) as a potential alternative has been drawing more and more attention. Therefore, we conducted a meta-analysis to quantitatively explore the association between TMB and outcomes of PD-1/PD-L1 inhibitions.

**Methods:** We searched eligible studies that evaluated the association between TMB and the outcomes of PD-1/PD-L1 inhibitions from PubMed, Embase, and Cochrane database up to October 2018. The primary endpoints were the progression-free survival (PFS) and the overall survival (OS) in patients with high TMB or low TMB. The pooled hazard ratios (HR) for PFS and OS were performed by Stata.

**Results:** In this analysis, a total of 2,661 patients from eight studies were included. Comparing PD-1/PD-L1 inhibitions to chemotherapy, the pooled HR for PFS and OS in patients with high TMB was 0.66 [95% confidence interval (CI) 0.50 to 0.88; *P* = 0.004] and 0.73 (95% CI 0.50 to 1.08; *P* = 0.114), respectively, while the pooled HR for PFS and OS in patients with low TMB was 1.38 (95% CI 0.82 to 2.31; *P* = 0.229) and 1.00 (95% CI 0.80 to 1.24; *P* = 0.970), respectively. Meanwhile, comparing patients with high TMB to patients with low TMB, the pooled HR for PFS in patients treated with PD-1/PD-L1 inhibitions was 0.47 (95% CI 0.35 to 0.63; *P* = 0.000). Patients with high TMB showed significant benefits from PD-1/PD-L1 inhibitions compared to patients with low TMB.

**Conclusion:** Despite the present technical and practical barriers, TMB may be a preferable biomarker to optimize the efficacy of PD-1/PD-L1 inhibitions.

## Introduction

Cancer is a serious health problem and is the major leading cause of death worldwide (Torre et al., 2015). On the basis of the statistics that was released by the International Agency for Research on Cancer in 2017, about 16.8 million new cancer cases and 6 million cancer deaths were reported each year (Siegel et al., 2017). It was shown that the 5-year survival rate for all tumor patients is only 67% (Tong et al., 2018). In the past decades, with the remarkable development of clinical therapies on oncology, based on the three traditional methods of cancer treatment (surgery, radiotherapy, and chemotherapy), plenty of novel treatments have been launched, including targeted therapy, interventional therapy, and immunotherapy.

As an innovative therapy, immunotherapy has become a hot spot in the field of cancer treatment. Currently, the main research directions of immunotherapy in the world are immune checkpoint inhibitors. The programmed cell death 1 (PD-1) or programmed cell death ligand 1 (PD-L1) inhibitions are the representative treatments involving immunotherapy. PD-1/PD-L1 inhibitions were licensed to treat a variety of cancers (Ribas and Wolchok, 2018). Currently, many studies have shown that PD-1/PD-L1 inhibitions could improve outcomes of cancer patients compared to chemotherapy (Borghaei et al., 2015; Herbst et al., 2016; Rittmeyer et al., 2017). However, the efficacy of PD-1/PD-L1 inhibitions varies from individual. Therefore, researchers are exploring some biomarkers to assess the efficacy of PD-1/PD-L1 inhibitions. In 2012, some studies showed that the level of PD-L1 expression in tumor tissue was related to the effect of treatment (Brahmer et al., 2012; Topalian et al., 2012).

U.S. Food and Drug Administration (FDA) recently approved pembrolizumab to treat advanced non-small cell lung cancer (NSCLC) and using the PD-L1 expression as a predictive biomarker to evaluate the outcomes of the treatment (FDA, 2015). The National Comprehensive Cancer Network (NCCN) guidelines of non-small cell lung cancer (version 1. 2017) also recommended all lung cancer patients to detect PD-L1 expression (National Comprehensive Cancer Network, 2016). Due to the recommendation, many previous meta-analyses had evaluated the value of PD-L1 detection, intending to prove the benefit of PD-L1 expression as a potential biomarker. However, there were some defects of PD-L1 expression as a biomarker (Topalian et al., 2016). For example, the level of PD-L1 expression in individual patients may change over time, or change by previous treatments and anatomical site; small biopsy specimens obtained by fine needle may miss some PD-L1 expression in tumors; antibodies used to detect PD-L1 expression have different affinities and specificities; different kits used to hold tumor samples may affect the detection of PD-L1 expression; detection platforms using different techniques may have different results on the level of PD-L1 expression. Meanwhile, some trials showed that patients with negative PD-L1 expression could have favorable outcomes (Motzer et al., 2015; Horn et al., 2017). Accordingly, PD-L1 expression may not be a preferable biomarker to predict the response of PD-1/PD-L1 inhibitions.

Tumor mutation burden (TMB) was another potential biomarker and was defined as the total number of somatic mutations per megabase or the nonsynonymous mutations in tumor tissues, including replacement and insertion deletion mutations. In some trials, the objective response rates of the PD-1/PD-L1 inhibitions were higher in patients with high TMB than in patients with low TMB (Carbone et al., 2017; Hellmann et al., 2018a; Hellmann et al., 2018b). However, as TMB remains a controversial biomarker for the patient selection and screening for the treatments of PD-1/PD-L1 inhibitions, we conducted a meta-analysis to quantitatively compare the efficacy of PD-1/PD-L1 inhibitions in patients with high TMB against patients with low TMB based on the most updated clinical evidence.

## Materials and Methods

This study was in accordance with the recommendations of the Cochrane Handbook for Systematic Reviews of Interventions and reported on the basis of the Preferred Reporting Items for Systematic Reviews and Meta-Analyses (PRISMA) statement guidelines.

### Literature Search

We collected the relevant studies published on PubMed, Embase, and Cochrane databases up to October 2018 without language restrictions. Considering that some studies may be unpublished, we also searched studies from the American Society of Clinical Oncology Annual Meeting (ASCO) and the European Society of Medical Oncology (ESMO).

We searched studies from these databases in all fields with “Nivolumab” OR “Opdivo” OR “ONO-4538” OR “Tecentriq” OR “MPDL-3280A” OR “RG-7446” OR “Pembrolizumab” OR “Keytruda” OR “Lambrolizumab” OR “MK-3475” OR “PEMBRO” OR “Durvalumab” OR “MEDI-4736” OR “Imfinzi” OR “Pidilizumab” OR “CT-011” OR “PD-1” OR “PD-L1” OR “PD-1/PD-L1” OR “programmed cell death 1” OR “programmed cell death ligand 1” AND “tumor mutation burden” OR “tumor mutation load” OR “TMB” OR “TML” as the keywords.

### Study Selection

We defined both inclusion and exclusion criteria in advance. Studies had to meet several inclusion criteria. Firstly, the levels of TMB in patients with tumor were examined. Secondly, the intervention was PD-1/PD-L1 inhibitions (nivolumab, pembrolizumab, atezolizumab, durvalumab, and avelumab). Different doses of the same drug and treatments with drug combination were included. Thirdly, the primary endpoints were the progression-free survival (PFS) measured by hazard ratios (HR) and the overall survival (OS) measured by HR in patients with high TMB or low TMB. Both randomized controlled trials and retrospective studies that met the above inclusion criteria were included.

Studies were excluded if they were review articles, perspective studies, cost-effectiveness analyses, commentaries, and irrelevant articles. Additionally, articles with incomplete data and published in different journals were also excluded. When the same clinical trial appeared in different articles, the latest or the most complete reporting study was included. All studies included in this meta-analysis were unique studies.

### Data Extraction and Risk of Bias Assessment

Data extraction and assessment were made independently by two different authors (JZ and TZ), and disagreement was solved by a discussion with another author (NW). The following information was extracted from each included trial: trial name/authors, year of publication, trial phase, line of treatment, type of cancer, experimental drugs, number of patients with high TMB and low TMB, PFS, and OS.

Among the eight included studies, three studies were randomized controlled trials, four studies were retrospective studies, and one study was the single-arm trial. The Cochrane Collaboration’s Tools (Higgins et al., 2011), Newcastle–Ottawa Scale (NOS) (Lo et al., 2014), and Methodological index for non-randomized studies (MINORS) (Slim et al., 2003) were applied to assess the risk of bias for randomized controlled trials, retrospective studies, and single-arm trial, respectively. In the Cochrane Collaboration’s Tools, seven items were scored as low, high, or unclear risk of bias, including random sequence generation, allocation concealment, blinding of participants and personnel, blinding of outcome assessment, incomplete outcome data, selective reporting, and other bias. In the NOS, three items were assessed including selection, comparability, and outcome. The total NOS scores were categorized into three groups: very high risk of bias (0 to 3 points), high risk of bias (4 to 6 points), and low risk of bias (7 to 9 points). In the MINORS, eight items were assessed including a clearly stated aim, inclusion of consecutive patients, prospective collection of data, endpoints appropriate to the aim of the study, unbiased assessment of the study endpoint, follow-up period appropriate to the aim of the study, loss to follow-up less than 5%, and prospective calculation of the study size, which were scored as 0 (not reported), 1 (reported but inadequate), or 2 (reported and adequate).

### Data Analysis

We defined 10 somatic mutations per megabase (Mut/Mb) [corresponding to approximately 150 nonsynonymous mutations according to Schumacher’s research (Schumacher and Schreiber, 2015)] as the cutoff. The patients with TMB at or above this cutoff were divided into the high TMB group, and the patients with TMB below this cutoff were divided into the low TMB group. The primary endpoints were the PFS and the OS in patients with high TMB or low TMB, which were measured by HR. Therefore, we derived the HR for death and corresponding 95% confidence intervals (CI) from each included trial, separately for patients with high TMB and low TMB. Then, the pooled HRs for PFS and OS were concluded by a meta-analysis.

We evaluated the statistical heterogeneity between different trials by using Cochrane’s *I* ^2^ statistics. If *I* ^2^ > 25%, the pooled HRs were calculated by the random effects models; otherwise, the pooled HRs were calculated by the fixed-effects models (Higgins et al., 2003). Subgroup analyses were conducted to explore the source of heterogeneity on the following selected subgroups: experimental drugs, underlying malignancy, method of TMB detection, and year of publication. Potential publication bias was assessed by the funnel plot and the Egger test (Egger et al., 1997). If a value of *P* < 0.05 or 95% CI did not contain 0, there was potential publication bias; otherwise, there was no potential publication bias. All analysis was performed by Stata version 14.0.

## Results

### Literature Search

**Figure 1:**
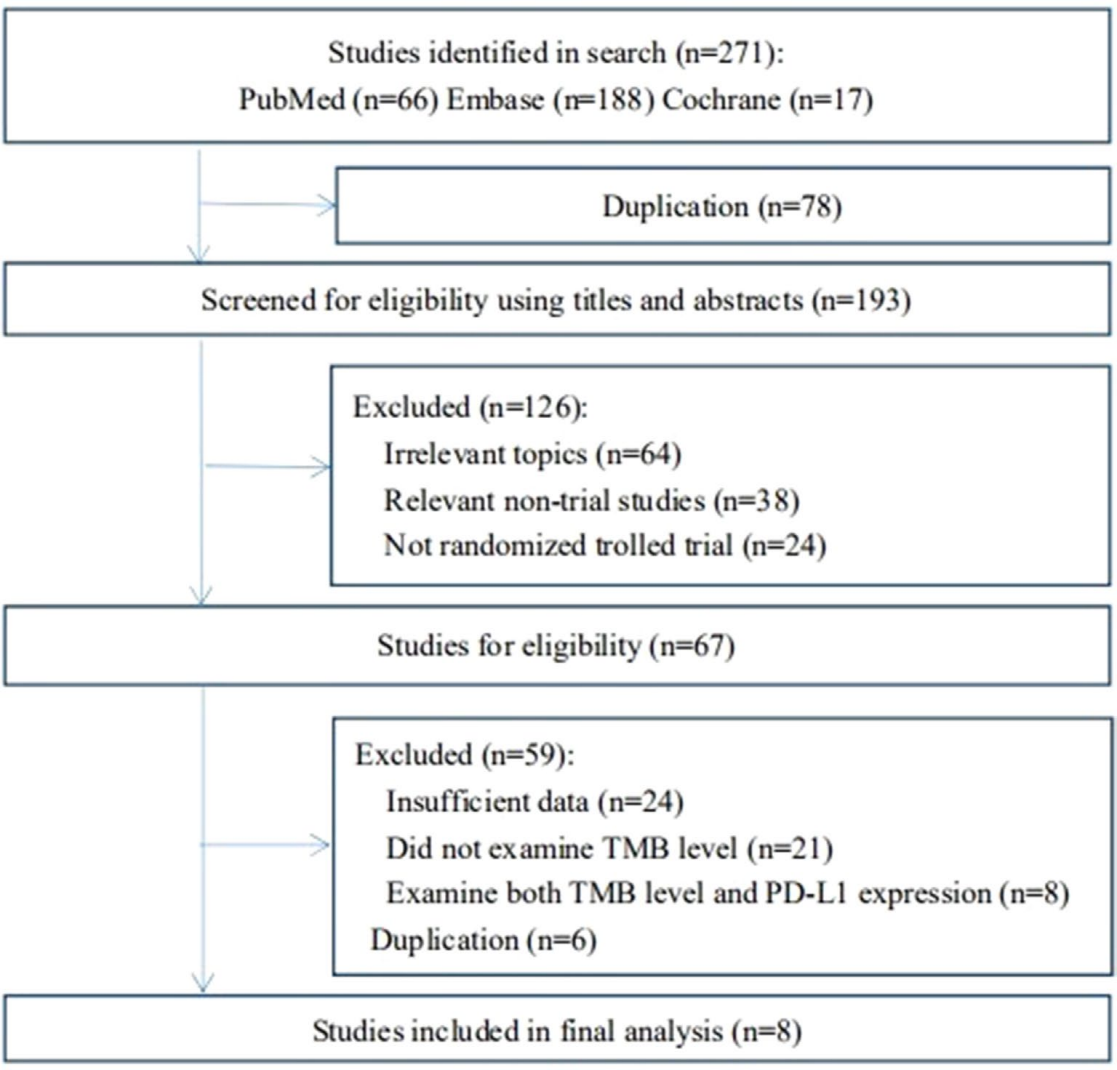
Flowchart diagram of selected trials included in this meta-analysis.

In the first searching strategy, there were 271 related articles in total. Due to duplication, 78 articles were excluded. After screening for eligibility using titles and abstracts, we removed 126 studies that did not meet the inclusion criteria. Then, we reviewed the full texts of the remaining 67 studies. Finally, eight studies were included (Rizvi et al., 2015; Carbone et al., 2017; Goodman et al., 2017; Kowanetz et al., 2017; Powles et al., 2018; Hellmann et al., 2018b; Hellmann et al., 2018c). The study selection flowchart is shown in **Figure 1**. Data from all included trials were obtained from published articles and their supplementary information.

### Study Characteristics

A total of 2,661 patients from eight trials were included in this analysis. The baseline characteristics and outcomes of each included trial are summarized in **Table 1**.

**Table 1:** The baseline characteristics of each included trial.

Study	Trial phase	Line of treatment	Underlying malignancy	Experimental drugs	Cutoff of TMB	Detection method	No. of patients				PFS	OS
							HTMB		LTMB			
							Intervention	Control	Intervention	Control		
Carbone D P et al. (2017)	III	1st	NSCLC	Nivolumab vs. chemotherapy	243 mutations	WES	47	60	111	94	HTMB: HR: 0.62 (95% CI: 0.38–1.00) LTMB: HR: 1.82 (95% CI:1.30–2.55)	HTMB: HR: 1.10 (95% CI: 0.64–1.88) LTMB: HR:0.99 (95% CI: 0.71–1.40)
Hellmann M D et al. (2018)	III	1st	NSCLC	Nivoluma + ipilimumab vs. chemotherapy	10 Mut/Mb	FM NGS	139	160	191	189	HTMB: HR: 0.58 (95% CI: 0.41–0.81) LTMB: HR: 1.07 (95% CI: 0.84–1.35)	NA
				Nivolumab vs. chemotherapy	13 Mut/Mb	FM NGS	71	79	NA	NA	HTMB: HR: 0.95 (95% CI: 0.64–1.40)	NA
Powles T et al. (2017)	III	1st	Urothelial carcinoma	Atezolizumab vs. chemotherapy	9.65 Mut/Mb	FM NGS	274		270		NA	HTMB: HR: 0·68 (95% CI: 0.51–0.90) LTMB: HR: 1.00 (95% CI: 0.75–1.32)
Kowanetz M et al. (2017)	NA	2nd	NSCLC	Atezolizumab vs. docetaxel	9.9 Mut/Mb	FM NGS	NA^a^		NA^a^		HTMB: HR: 0.49 (95% CI: 0.25–0.93)	HTMB: HR: 0.48 (95% CI: 0.23–1.04)
		1st	NSCLC	Atezolizumab	9 Mut/Mb	FM NGS	NA^b^		NA^b^		HR: 0.58 (95% CI: 0.36–0.94)	HR: 0.79 (95% CI: 0.39–1.58)
		2nd	NSCLC	Atezolizumab	9.9 Mut/Mb	FM NGS	NA^c^		NA^c^		HR: 0.64 (95% CI: 0.50–0.80)	HR: 0.87 (95% CI: 0.65–1.16)
Rizvi N A et al. (2015)	NA	NA	NSCLC	Pembrolizumab	200 mutations	WES	17		17		HR: 0.19 (95% CI: 0.08–0.47)	NA
Hellmann M D et al. (2018)	NA	NA	NSCLC	Nivolumab + ipilimumab	158 mutations	WES	37		38		HR: 0.41 (95% CI: 0.23–0.73)	NA
CheckMate 817	III	1st	NSCLC	Nivolumab + ipilimumab	10 Mut/Mb	FM NGS	73		78		HR: 0.60 (95% CI: 0.40–0.90)	NA
Goodman A M et al. (2017)	NA	NA	Diverse cancers^d^	Immunotherapy	20 Mut/Mb	FM NGS	38		113		HR:0.34 (95% CI: 0.23–0.50)	HR:0.33 (95% CI: 0.19–0.58)

a The total number of patients in HTMB and LTMB groups is 92.

b The total number of patients in HTMB and LTMB groups is 102.

c The total number of patients in HTMB and LTMB groups is 371.

d Cancers included the following: melanoma (n = 52), non-small cell lung cancer (n = 36), head and neck (n = 13), cutaneous squamous cell carcinoma (n = 8), renal cell carcinoma (n = 6), colon adenocarcinoma (n = 5), bladder transitional cell carcinoma (n = 4), breast cancer (n = 3), hepatocellular carcinoma (n = 3), sarcoma (n = 3), thyroid cancer (n = 3), basal cell carcinoma (n = 2), cervical cancer (n = 2), Merkel cell carcinoma (n = 2), ovarian carcinoma (n = 2), unknown primary squamous cell carcinoma (n = 2), adrenal carcinoma (n = 1), appendix adenocarcinoma (n = 1), pleural mesothelioma (n = 1), prostate cancer (n = 1), and urethral squamous cell carcinoma (n = 1).

NSCLC, non-small cell lung cancer; Mut, mutations; Mb, megabase; WES, whole exome sequencing; NGS, next-generation sequencing; FM, Foundation Medicine; NA, not available; HTMB, high tumor mutation burden; LTMB, low tumor mutation burden; HR, hazard ratio; PFS, progression-free survival; OS, overall survival; CI, confidence interval.

Among eight included studies, six studies were conducted in patients with non-small cell lung cancer, one study was conducted in patients with urothelial carcinoma, and one study was conducted in patients with diverse cancers. According to the subjects in the intervention arm, three studies received nivolumab plus ipilimumab, two studies received atezolizumab and nivolumab, and one study received each of pembrolizumab and diverse immune checkpoint inhibitors.

The quality of the included studies was generally moderate to good (**Appendix Tables 1–3 in Supplementary Material**).

### Efficacy Comparison for PD-1/PD-L1 Inhibitions versus Chemotherapy According to the Level of TMB

**Figure 2:**
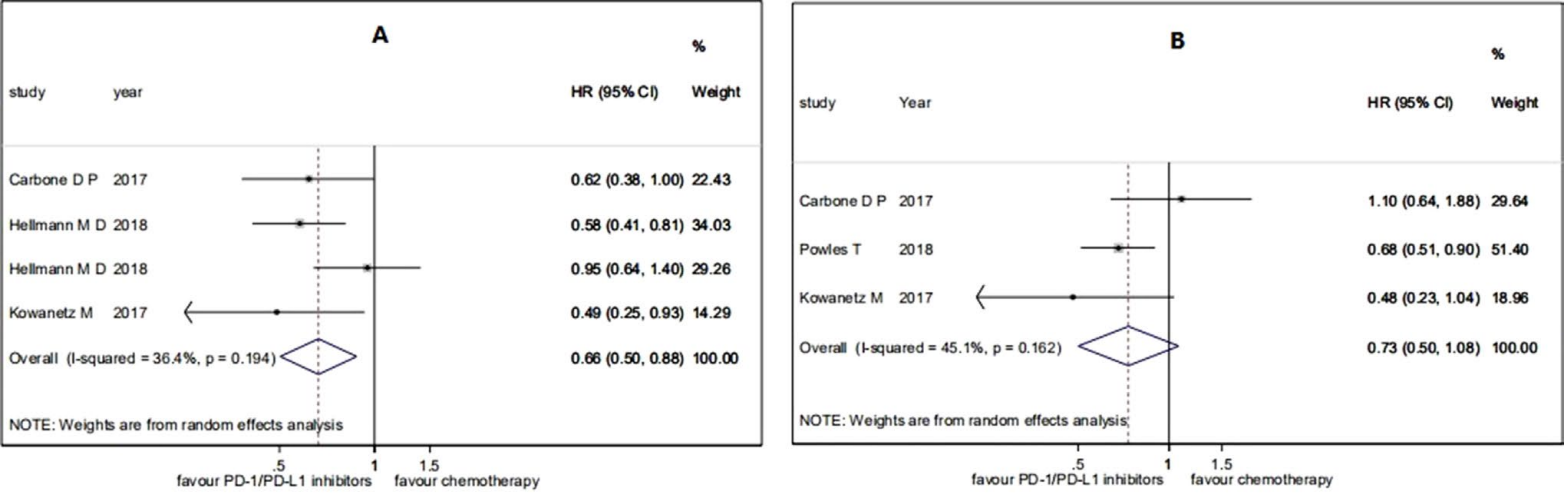
Forest plots of **(A)** HR of progression-free survival (PFS) and **(B)** HR of overall survival (OS) in patients with high tumor mutation burden (TMB) assigned to treat with immunotherapy versus chemotherapy. HR, hazard ratio.

**Figure 3:**
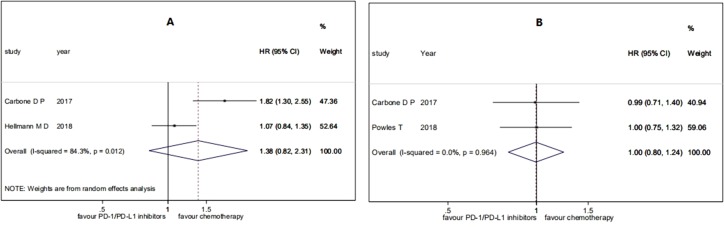
Forest plots of **(A)** HR of PFS and **(B)** HR of OS in patients with low TMB assigned to treat with immunotherapy versus chemotherapy. HR, hazard ratio.

For the patients with high TMB, the pooled HR for PFS was 0.66 (95% CI 0.50 to 0.88; *P* = 0.004), and the heterogeneity was observed (*I* ^2^ = 36.4%, *P* = 0.194), while the pooled HR for OS was 0.73 (95% CI 0.50 to 1.08; *P* = 0.114), and the heterogeneity was observed (*I* ^2^ = 45.1%, *P* = 0.162) (see **Figure 2**). For the patients with low TMB, the pooled HR for PFS was 1.38 (95% CI 0.82 to 2.31; *P* = 0.229), and the heterogeneity was observed (*I* ^2^ = 84.3%, *P* = 0.012), while the pooled HR for OS was 1.00 (95% CI 0.80 to 1.24; *P* = 0.970), and no heterogeneity was observed (*I* ^2^ = 0.0%, *P* = 0.964) (see **Figure 3**). The subgroup analyses for patients with high TMB assigned to treat with immunotherapy versus chemotherapy showed that the heterogeneity was mainly caused by the type of experimental drugs (**Appendix Figure 1 in Supplementary Material**).

In a summary, among the patients treated with PD-1/PD-L1 inhibitions versus chemotherapy, patients with high TMB had significant benefits on PFS and patients with low TMB had no significant benefits on PFS, while both patients with high TMB and patients with low TMB had no significant benefits on OS.

### Efficacy Comparison for PD-1/PD-L1 Inhibitions in Patients With High TMB versus Patients With Low TMB

**Figure 4:**
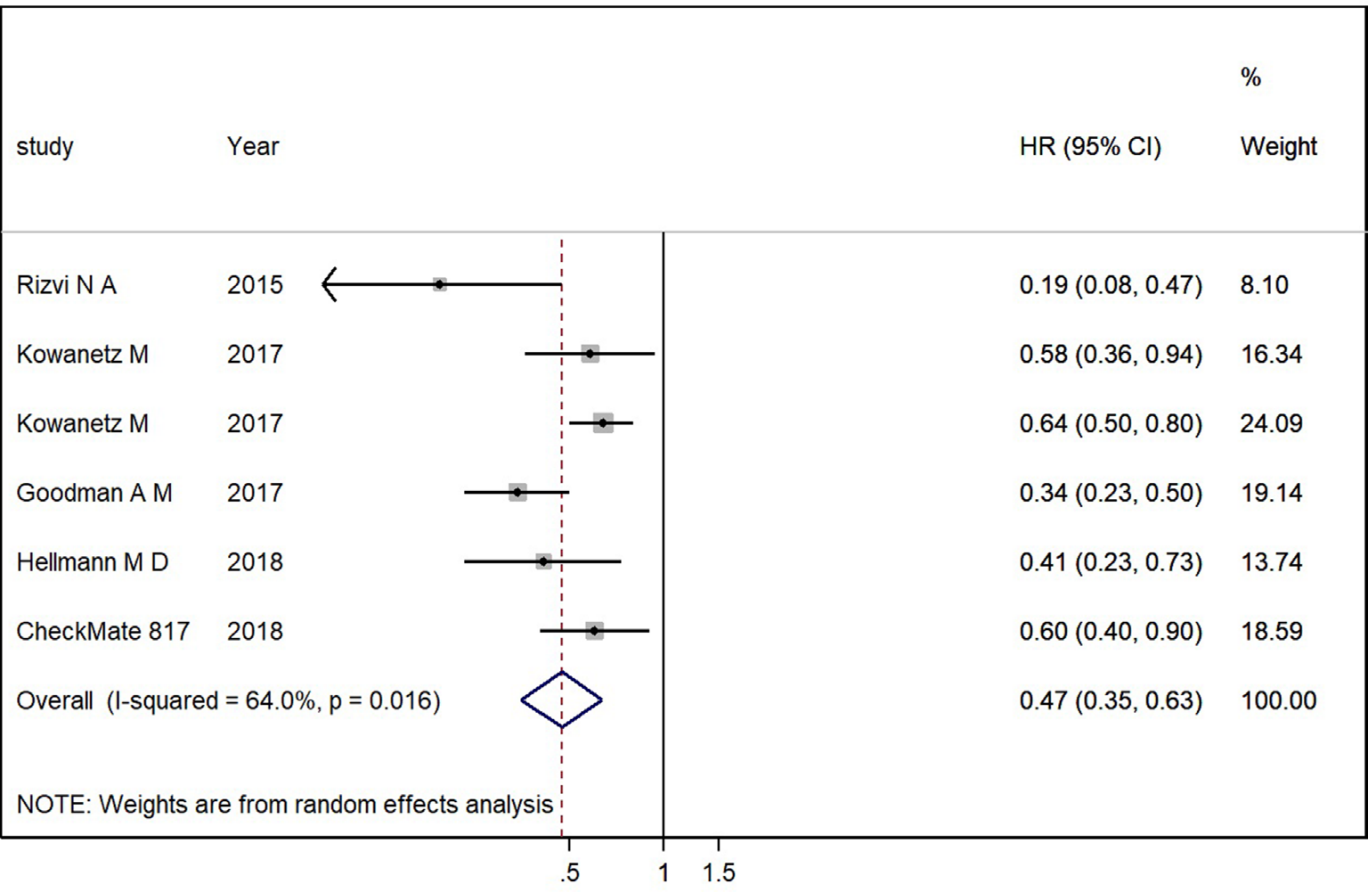
Forest plots of HR of PFS in patients with high TMB versus patients with low TMB. HR, hazard ratio.

For the efficacy of PD-1/PD-L1 inhibitions on patients with high TMB versus patients with low TMB, the pooled HR for PFS was 0.47 (95% CI 0.35 to 0.63; *P* = 0.000), and the heterogeneity was observed (*I* ^2^ = 64.0%, *P* = 0.016) (see **Figure 4**). The subgroup analyses for patients with high TMB versus patients with low TMB showed that the heterogeneity was mainly caused by the type of experimental drugs (**Appendix Figure 2 in Supplementary Material**).

Overall, the analysis showed that patients with high TMB had significant benefits on PFS compared to patients with low TMB when they were treated with PD-1/PD-L1 inhibitions.

### Sensitivity Analysis

Due to substantial heterogeneity, we moved out two studies (Rizvi et al., 2015; Goodman et al., 2017) to evaluate the influence of individual trial on the primary endpoints. One retrospective study was excluded for the big difference in the number of patients between high TMB and low TMB groups. Another study only involved 34 people to unravel the genomic determinants of response to immune checkpoint inhibitors. After removing two studies, we could also get similar efficacy results in patients with high TMB and low TMB, and the heterogeneity was not observed (**Appendix Figure 3 in Supplementary Material**).

### Publication Bias

The funnel plots did not show substantial asymmetry (**Appendix Figures 4–8 in Supplementary Material**). The Egger linear regression test also indicated no evidence of publication bias.

## Discussion

Based on the previous qualitative study (Chan et al., 2018), our quantitative research showed that patients with high TMB had significant benefits on PFS compared to patients with low TMB when they were treated with PD-1/PD-L1 inhibitions, while both patients with high TMB and patients with low TMB had no significant benefits on OS when they were assigned to receive PD-1/PD-L1 inhibitions versus chemotherapy, which may result from the limited follow-up duration and the treatment crossover between intervention and control groups. Accordingly, TMB could be a preferable biomarker for the selection of the most appropriate patients treated by PD-1/PD-L1 inhibitions compared to PD-L1 expression.

As to the exploration of optimal cutoff value, based on the Foundation Medicine official reports, the levels of TMB were divided into three groups: low (1 to 5 Mut/Mb), intermediate (6 to 19 Mut/Mb), and high (≥20 Mut/Mb) (Goodman et al., 2017). However, in clinical practice, around 10 Mut/Mb or 150 mutations were more frequently set as the cutoff to divide patients into high and low expression of TMB (Kowanetz et al., 2017; Powles et al., 2018; Ramalingairn et al., 2018; Hellmann et al., 2018b; Hellmann et al., 2018c). In addition, using lower cutoff would increase the risk of false positives on the detection results of TMB (Chan et al., 2018), whereas using a higher cutoff with 15 Mut/Mb and 15.8 Mut/Mb did not improve efficacy in NSCLC patients from PD-1/PD-L1 inhibitions (Kowanetz et al., 2017; Ramalingairn et al., 2018). Therefore, we defined 10 Mut/Mb or 150 mutations as the cutoff and found out it was an ideal cutoff value to distinguish NSCLC patients from high TMB and low TMB. As to the blood-based TMB detection, 16 Mut/Mb was considered as the best cutoff (Gandara et al., 2018).

However, TMB detection was far from perfect. Firstly, one study showed that TMB level may decrease by the storage time causing inaccurate detection results of TMB (Chen et al., 2018). Secondly, the levels of TMB varied from detection methods. There were two methods to detect TMB, including whole exome sequencing (WES) and next-generation sequencing (NGS). Each method captured different types of mutations and had different capture regions, causing inconsistent TMB detection results for the same sample. Both methods have advantages and disadvantages. Though WES was accepted as a gold standard for TMB detection, it has not been used as a clinical tool to predict the responses of immune checkpoint inhibitors considering its high cost and it being time-consuming (Johnson et al., 2016). NGS was more convenient than WES, but the number of tumor mutations detected by NGS is required to be converted into the number of missense mutations determined by WES. Thirdly, both methods required a large number of tumor tissue samples, which was an invasive trauma, and tumors of some patients were too small to sample. To overcome this weakness, a novel, less time-consuming, more convenient blood-based method of TMB detection was introduced, with the intention of replacing the tissue-based method (Gandara et al., 2018).

According to the most updated NCCN guideline of non-small cell lung cancer, TMB was considered as an emerging biomarker for treatments with nivolumab and nivolumab plus ipilimumab (National Comprehensive Cancer Network, 2018). In our analysis, TMB detection may also be recommended for more treatments such as atezolizumab and pembrolizumab to screen the most appropriate patients. Meanwhile, we suggested a cutoff of 10 Mut/Mb or 150 mutations to divide NSCLC patients into those high TMB and those with low TMB. In addition, more tumor types such as urothelial carcinoma, melanoma, cutaneous squamous cell carcinoma, renal cell carcinoma, and lung large cell carcinoma may also use TMB as a biomarker to screen the preferable patients.

This study also has some limitations. First of all, most of our included studies focused on NSCLC; our conclusion should be taken cautiously when extrapolated to other tumors. Then, there was significant heterogeneity between the included studies, especially in the comparison of PD-1/PD-L1 inhibitions versus chemotherapy in the group of low-TMB patients. There were only two studies involving PFS and OS in patients with low TMB. As shown in the Results section for the subgroup analyses of the patients with high TMB, the heterogeneity was mainly caused by the type of experimental drugs. Hence, there was heterogeneity in two studies involving different experimental drugs. Besides, baseline characteristics of these two studies differed in race, gender, age, smoking history, etc., which would also result in heterogeneity. Therefore, our results should be confirmed by a larger population with different baseline characteristics. Furthermore, the efficacy of combined detection of TMB and PD-L1 expression should be further investigated and a cost-effectiveness analysis is needed given the high cost of TMB detection despite its significant strength in selecting appropriate patients for PD-1/PD-L1 inhibitions.

## Conclusions

Patients with high TMB have significant benefits from PD-1/PD-L1 inhibitions compared to patients with low TMB. Despite the present technical and practical barriers, TMB may be a preferable biomarker to screen the most appropriate patients treated with PD-1/PD-L1 inhibitions.

## Author Contributions

JZ and TZ led the development of this meta-analysis and contributed to the writing of the draft manuscript; JJ and NW contributed to the study design and methodology; JZ, JHL and JML carried out the literature search and assessments for the risk of bias; WL and WH reviewed, analyzed, and interpreted the data. All authors contributed to the interpretation of the results and approved the final version.

## Funding

This study was supported by the National Natural Science Foundation of China (grant no. 71704064), the Natural Science Foundation of Guangdong Province, China (grant no. 2017A030310174), and the Fundamental Research Funds for the Central Universities (grant no. 21616324).

## Conflict of Interest Statement

The authors declare that the research was conducted in the absence of any commercial or financial relationships that could be construed as a potential conflict of interest.
