# Supplementary material for: Association Between Tumor Mutation Burden (TMB) and Outcomes of Cancer Patients Treated With PD-1/PD-L1 Inhibitions: A Meta-Analysis
Source: Front Pharmacol. 2019 Jun 14;10:673. doi: 10.3389/fphar.2019.00673 (PMC6587434; doi:10.3389/fphar.2019.00673)
Supplement: Supplementary file 1 [file DataSheet_1.pdf]

Supplementary Material

1 Supplementary Tables

Supplementary Table 1. The Cochrane collaboration's tool for assessing risk of bias of the included studies.

Study	Random sequence generation	Allocation concealment	Blinding of participants and personnel	Blinding of outcome assessment	Incomplete outcome data	Selective reporting	Other bias
Carbone D P et al.(2017)	Low	Low	High	Low	Low	Low	Low
Powles T et al.(2017)	Low	Low	High	Low	Low	Low	Low
Hellmann M D et al.(2018)	Low	Low	High	Low	Low	Low	Low

Supplementary Table 2. The Newcastle-Ottawa Scale (NOS) assessment for risk of bias of the included studies.

Study	Selection	Comparability	Outcome	Total score*
Rizvi N A et al.(2015)	2	1	1	4
Kowanetz M et al.(2017)	3	1	2	6
Goodman A M et al.(2017)	3	1	3	7
Hellmann M D al.(2018)	3	1	3	7

*NOS points : 0 to 3 : very high risk of bias; 4 to 6: high risk of bias; 7 to 9: low risk of bias.

Supplementary Table 3. Methodological index for non-randomized studies for risk of bias.

Items	Score*
1. A clearly stated aim	2
2. Inclusion of consecutive patients	2
3. Prospective collection of data	2
4. Endpoints appropriate to the aim of the study	2
5. Unbiased assessment of the study endpoint	0
6. Follow-up period appropriate to the aim of the study	2
7. Loss to follow up less than 5%	1
8. Prospective calculation of the study size	2

*This method assessed risk of bias of CheckMate 817. The items are scored 0 (not reported), 1 (reported but inadequate) or 2 (reported and adequate).

2 Supplementary Figures

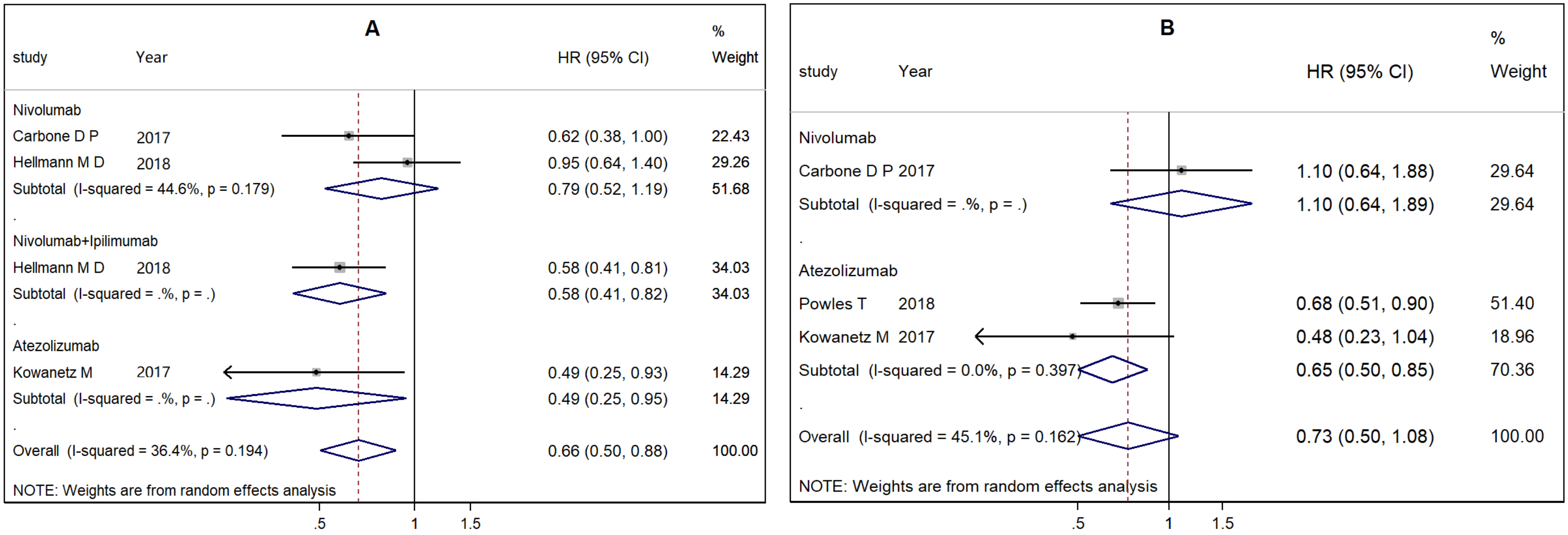

Supplementary Figure 1. Subgroup analysis for (A) PFS; (B) OS in patients with high TMB assigned to treat with immunotherapy versus chemotherapy. HR: Hazard Ratio.

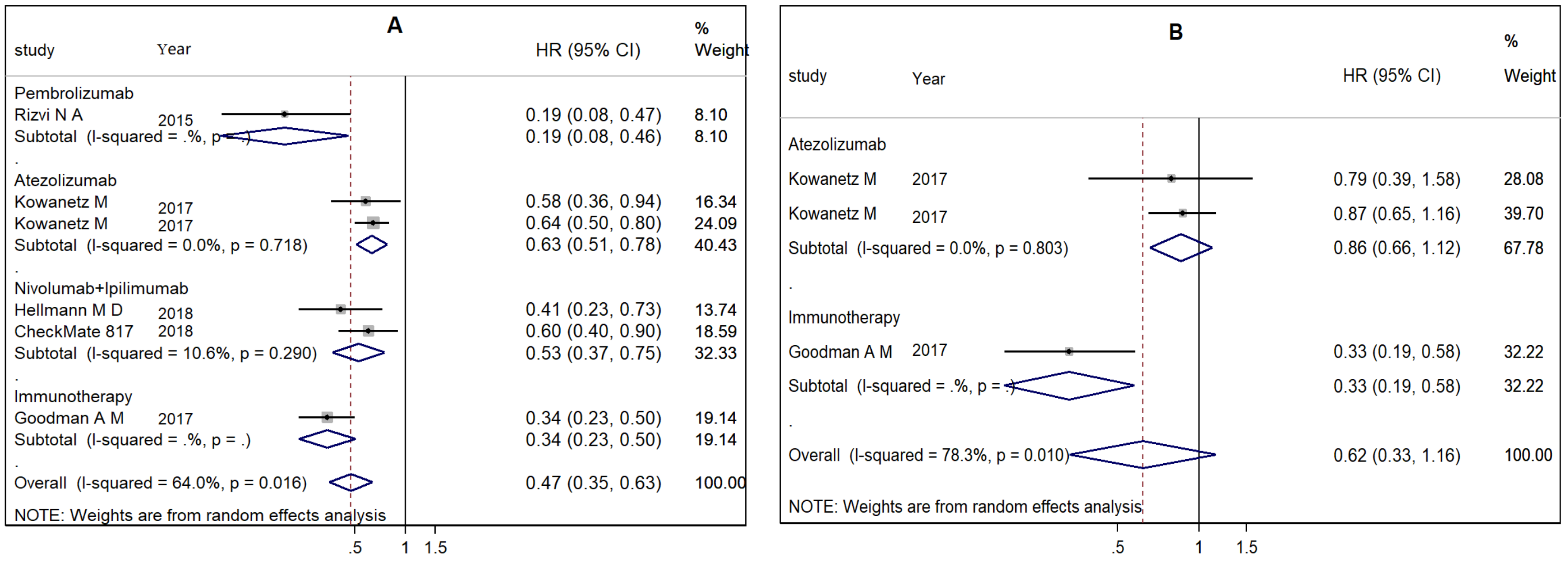

Supplementary Figure 2. Subgroup analysis for (A) PFS; (B) OS in patients with high TMB versus patients with low TMB. HR: Hazard Ratio.

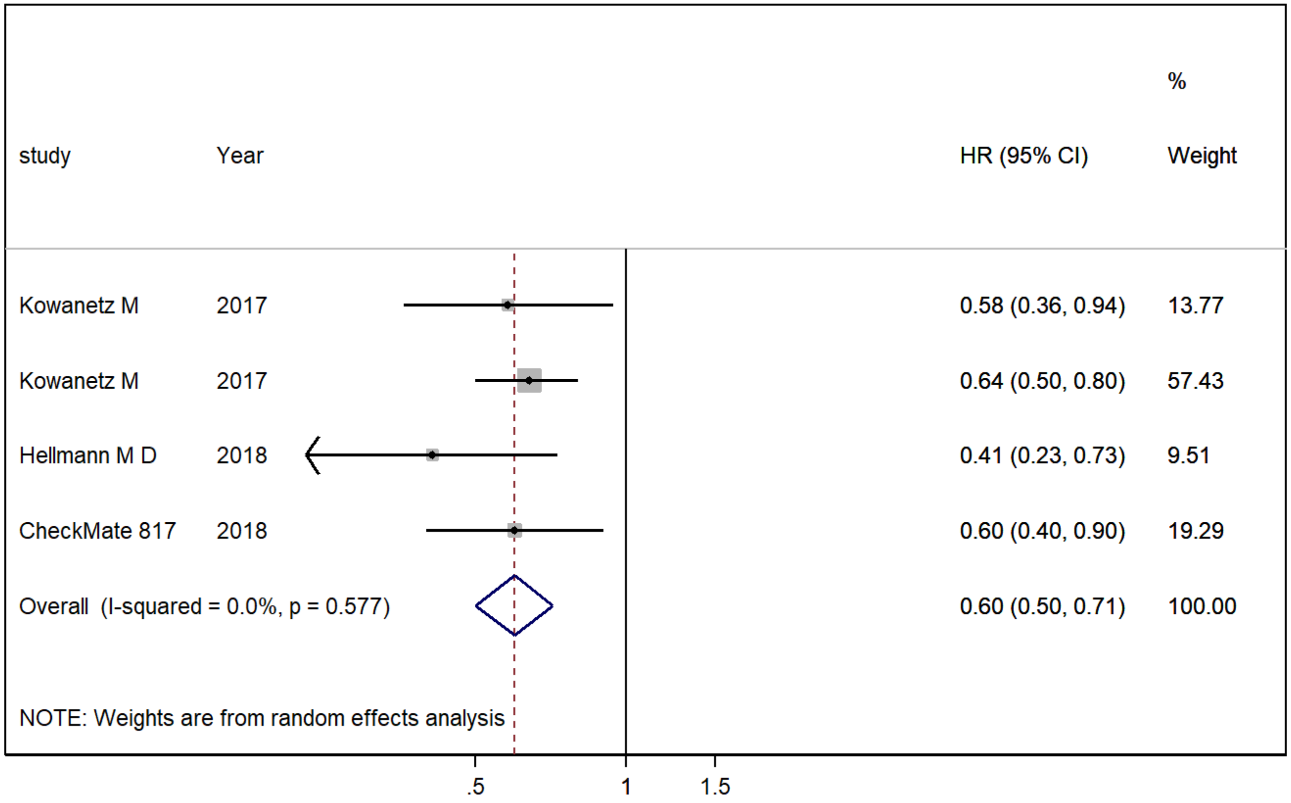

Supplementary Figure 3. Sensitivity analysis for PFS in patients with high TMB versus patients with low TMB. HR: Hazard Ratio.

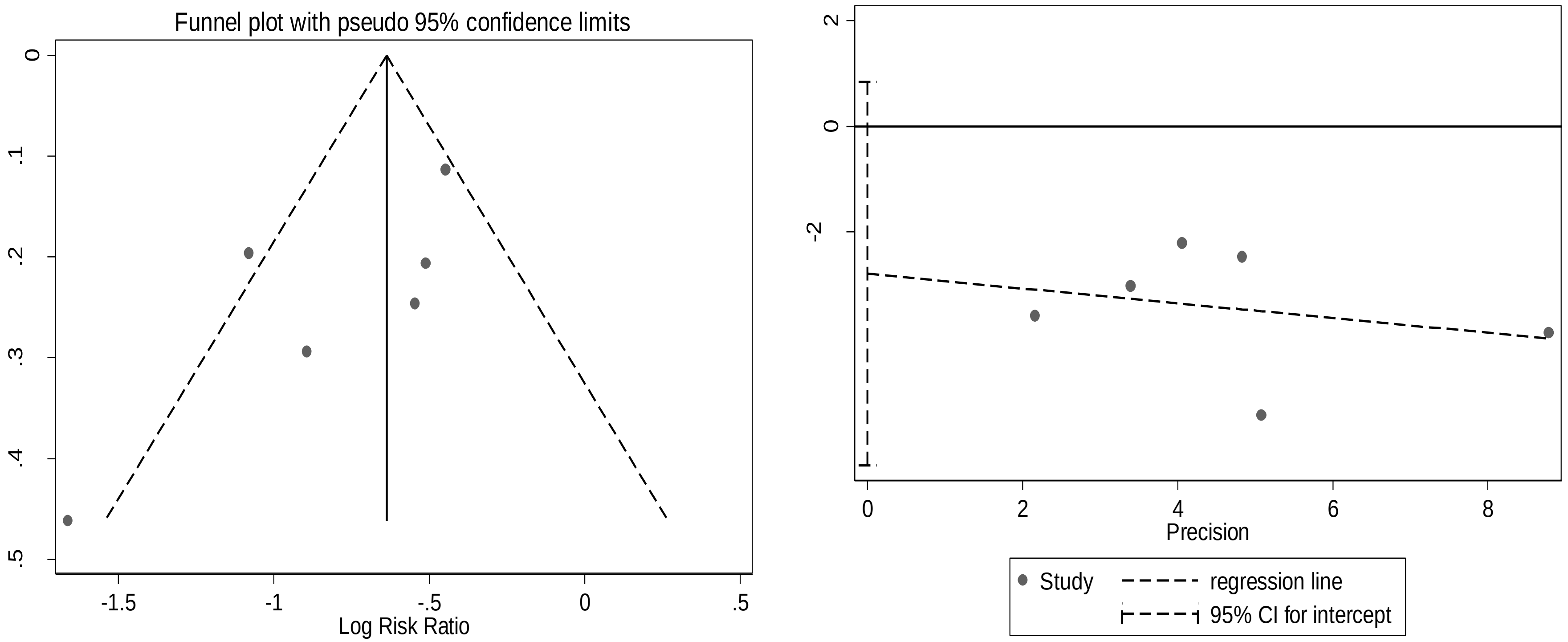

A B

Supplementary Figure 4. Funnel plot (A), and Egger plot (B) for publication bias for PFS in patients with high TMB versus patients with low TMB.

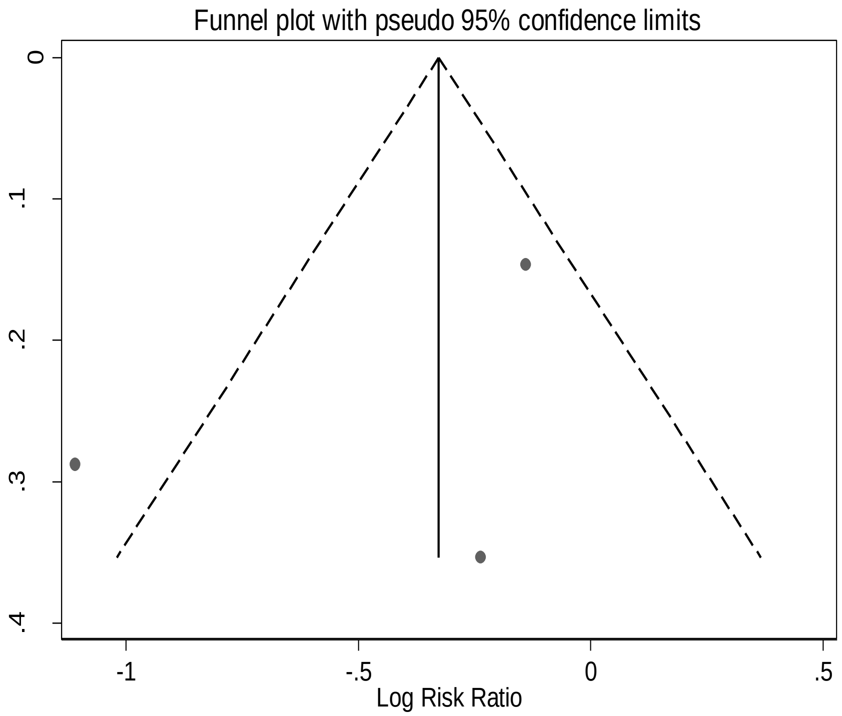

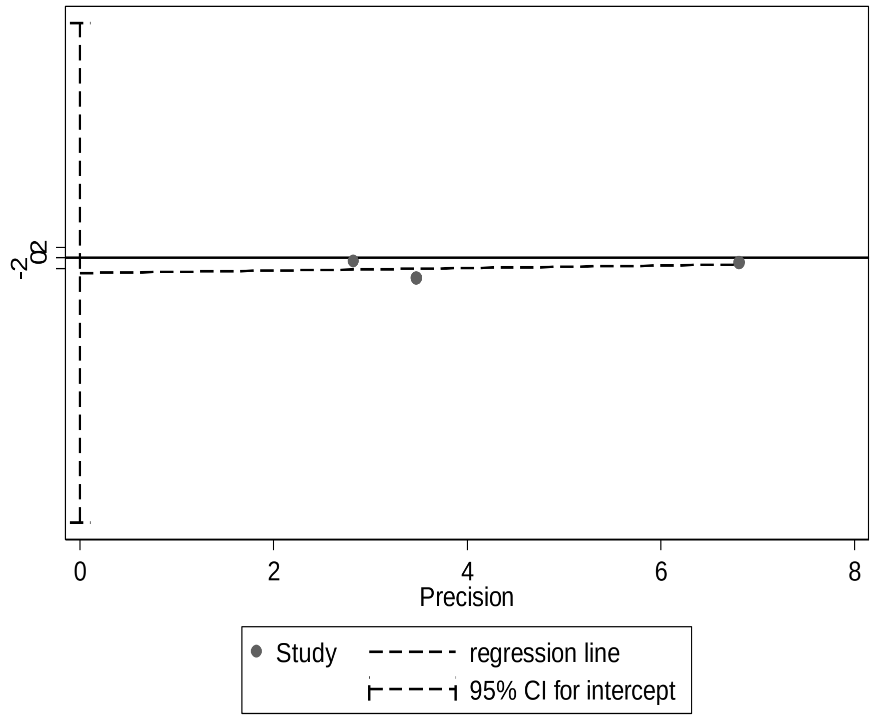

A B

Supplementary Figure 5. Funnel plot(A), and Egger plot(B) for publication bias for OS in patients with high TMB versus patients with low TMB

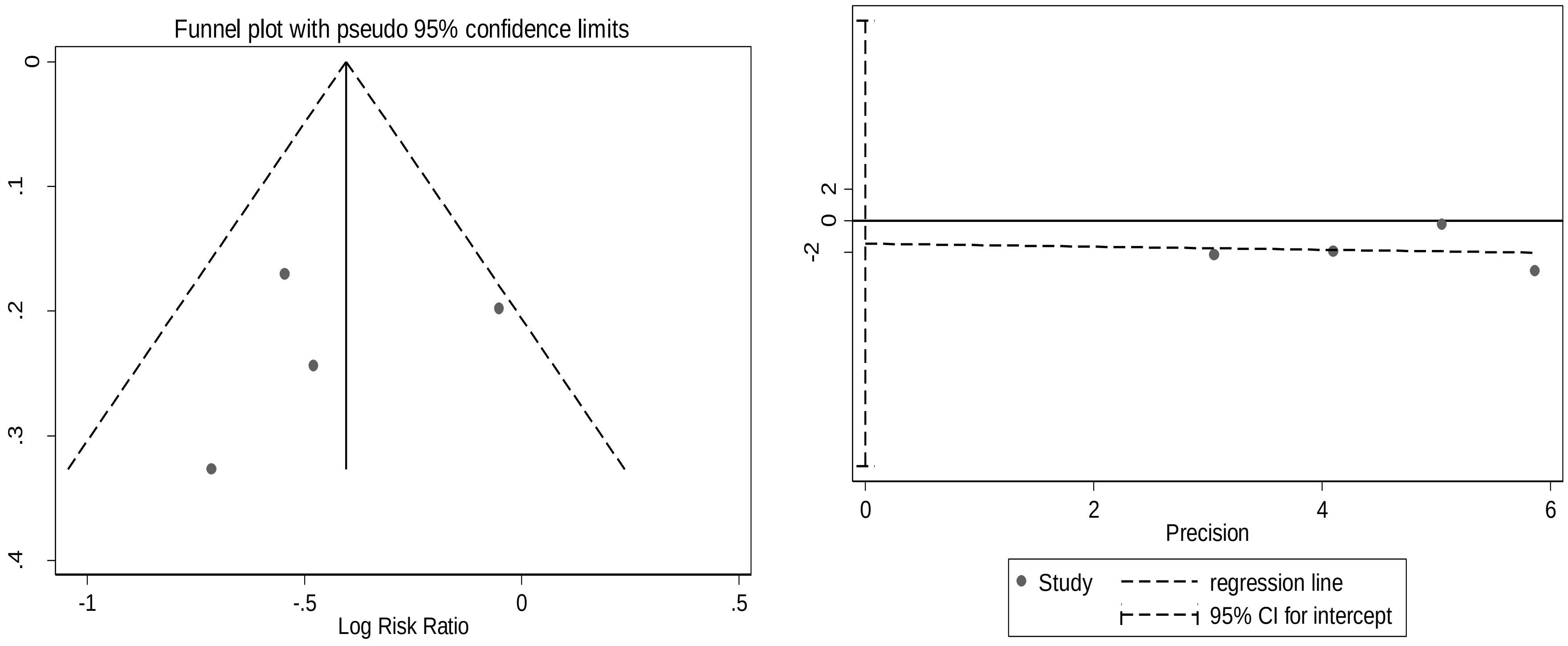

A B

Supplementary Figure 6. Funnel plot(A), and Egger plot(B) for publication bias for PFS in patients with high TMB assigned to treat with immunotherapy versus chemotherapy.

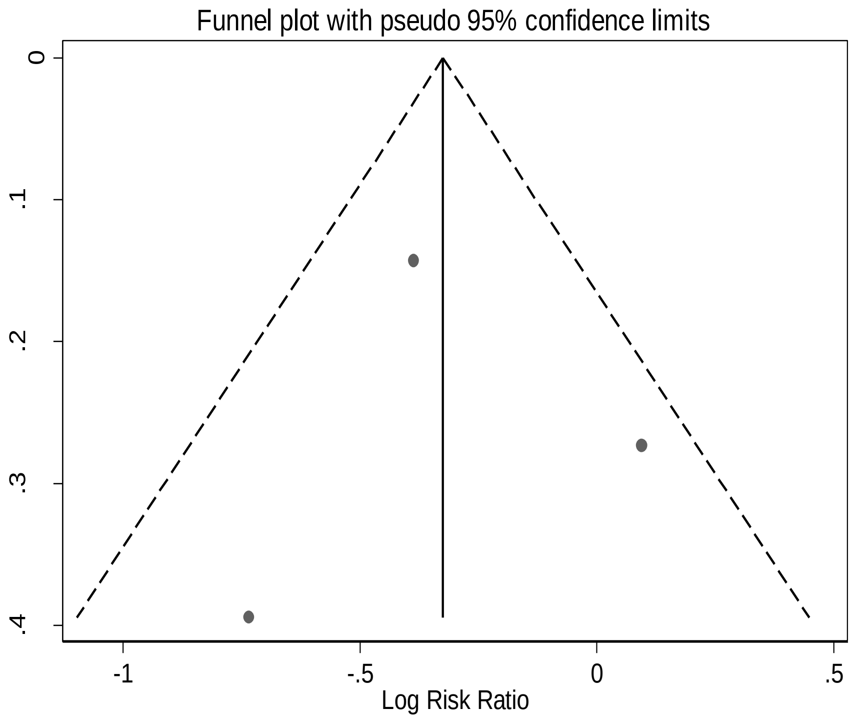

A

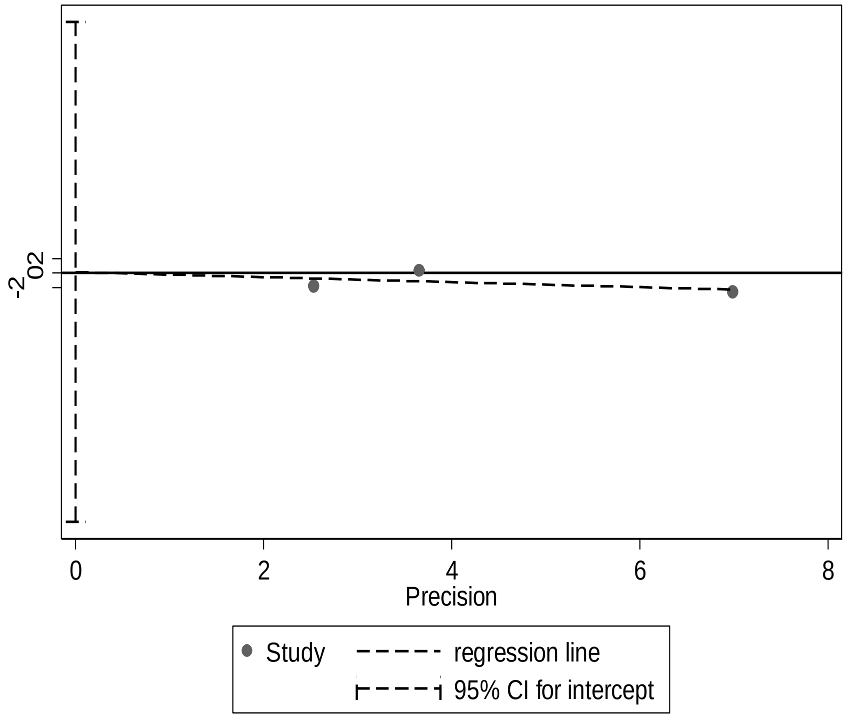

B

Supplementary Figure 7. Funnel plot (A), and Egger plot(B) for publication bias for OS in patients with high TMB assigned to treat with immunotherapy versus chemotherapy.

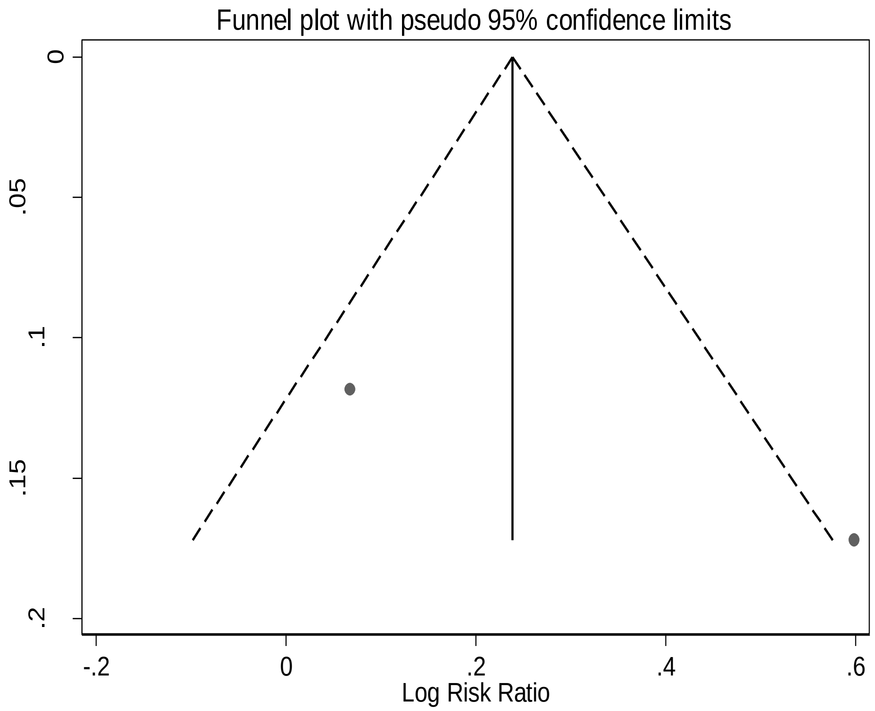

A

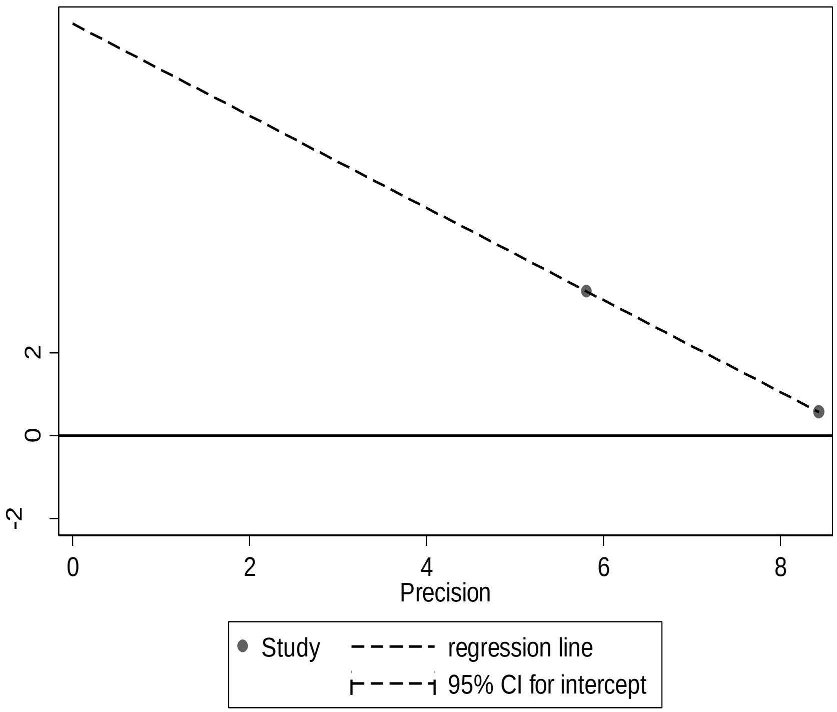

B

Supplementary Figure 8. Funnel plot (A), and Egger plot (B) for publication bias for PFS in patients with low TMB assigned to treat with immunotherapy versus chemotherapy.
